# Non-Enzymatic Template-Directed Recombination of RNAs

**DOI:** 10.3390/ijms10041788

**Published:** 2009-04-21

**Authors:** Sergey Y. Nechaev, Alexei V. Lutay, Valentin V. Vlassov, Marina A. Zenkova

**Affiliations:** Institute of Chemical Biology and Fundamental Medicine, Siberian Branch of Russian Academy of Sciences, 8 Lavrentiev Avenue, Novosibirsk 630090, Russian Federation; E-Mails: lutay_av@niboch.nsc.ru (A.L.); valentin.vlassov@niboch.nsc.ru (V.V.); marzen@niboch.nsc.ru (M.Z.)

**Keywords:** RNA, non-enzymatic recombination, non-enzymatic ligation, RNA bulge loops, RNA internal loops, RNA world, origin of life

## Abstract

RNA non-enzymatic recombination reactions are of great interest within the hypothesis of the “RNA world”, which argues that at some stage of prebiotic life development proteins were not yet engaged in biochemical reactions and RNA carried out both the information storage task and the full range of catalytic roles necessary in primitive self-replicating systems. Here we report on the study of recombination reaction occuring between two 96 nucleotides (nts) fragments of RNAs under physiological conditions and governed by a short oligodeoxyribonucleotide template, partially complementary to sequences within each of the RNAs. Analysis of recombination products shows that ligation is predominantly template-directed, and occurs within the complementary complex with the template in “butt-to-butt” manner, in 1- or 3- nts bulges or in 2–3 nts internal loops. Minor recombination products formed in the template-independent manner are detected as well.

## 1. Introduction

An evolutionary approach to the explanation of origin and development of life infers the implementation of two essential processes: the formation of diversity of some kind of objects and the mechanisms of selection of those which are the most adapted to survival under environmental conditions. The hypothesis of the “RNA world” suggests that RNA was able to store information, reproduce itself and evolve under prebiotic conditions without participation of any enzymes [1]. Supposedly, recombination events between short oligomers of RNA, formed in polymerization reactions, led to the increase of diversity of nucleotide sequences of RNA. Further development of life demanded an occurrence of mechanisms for molecular selection and evolution. For instance, some studies suggest that montmorillonite could play this role *via* absorbing the substrates of recombination reaction in its interlayers [2,3]. It is entirely possible that posterior stages of the “RNA world” evolution were signified with substitution of minerals by RNA in the capacity of a template, and complementary interactions became the basis for ancient mechanisms of selection [4].

Consecutive cleavage and ligation reactions of RNA are one of the foremost ways leading to their recombination. Both reactions involve transesterification, which implies the involvement of 2′-OH group of ribose in the cleavage site for the ester bond transfer. This is the main reason for most likely participation of RNA rather than DNA in the evolution under prebiotic conditions. Transesterification occurs spontaneously at alkaline pH, and can be accelerated significantly in the presence of organic bases or divalent metal ions, such as Mn^2+^, Mg^2+^, Pb^2+^, etc. [5–7]. Along with a structural role, metal ions promote rearrangement of electronic density in the cleavage or ligation site *via* coordination to 2′-, 3′- and 5′- oxygens of the phosphodiester bond [8]. In chemical terms, ligation reaction is reverse to cleavage; however, RNA fragments undergoing ligation may differ from those formed at the cleavage step, and thereby ensure the formation of novel RNA sequences. Occurrence of such reactions was first reported by Chetverin *et al*., who investigated random non-enzymatic recombination reactions in a pool of RNA within molecular colonies, further amplified and detected using Qβ replicase [9,10].

In a previous paper, we reported on the formation of new RNA sequences as a result of consecutive template-directed cleavage and ligation reactions of short RNA oligonucleotides [11]. In our earlier works with chimeric DNA/RNA oligonucleotides, the chemical mechanism for a new phosphodiester bond formation was investigated. It was shown that reaction intermediates possessed 2′,3′- cyclophosphate and 5′-OH group, and the ligation proceeded through the mechanism of intermolecular transesterification [12].

In the present article, we report the studies of consecutive cleavage/ligation reaction of two 96 nts long RNA fragments, catalyzed by Mg^2+^. The reaction was performed in the presence of complementary oligodeoxyribonucleotide, which governed the recombination within selected RNA sites. The products of recombination were amplified, isolated and their nucleotide sequences were determined. On the basis of sequencing data, the conclusions on the characteristic properties and mechanisms of recombination events were made.

## 2. Results and Discussion

### 2.1. Description of model system and reaction scheme

For investigation of the non-enzymatic RNA recombination reaction, occurring through cleavage and ligation steps, we used two RNA fragments as substrates. Initially these molecules were exposed to the action of a cleaving agent, Mg^2+^, for inducing the rupture of phosphodiester bonds within “fragile” sites of RNAs. As a result of the cleavage step, we obtained a pool of RNA fragments comprising 2′,3′-cyclophosphates, which can be considered as a set of activated substrates for the ligation step. Recombination reaction was performed in the presence of short DNA template (ONtemplate), which was expected to promote approaching of two selected fragments originating from different RNAs for their ligation. We suggested that use of the template would ensure the preferable ligation in butt-to-butt manner, within a complementary pseudoduplex of RNAs and the template.

**Figure 1.**
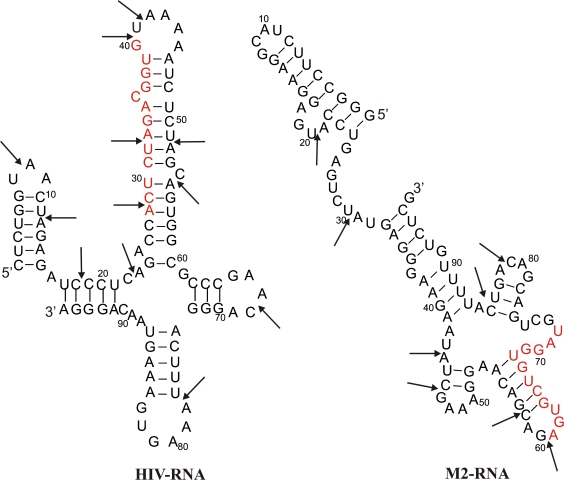
Fragments of HIV-RNA and M2-RNA, used as substrates in the non-enzymatic cleavage/ligation (recombination) reaction. Regions of RNAs, complementary to the oligodeoxyribonucleotide template, are highlighted in red. Arrows show internucleotide linkages most susceptible to cleavage (“fragile” sites) [13,16].

The RNA substrates used in the study are 96 nts fragment of HIV-1 genomic RNA, comprising viral primer binding site (HIV-RNA) and 96 nts fragment of Influenza virus A/WSN33/H1N1/genomic RNA, encoding M2 protein (M2-RNA). The major criterion for RNA fragments choice was the absence of homology between their sequences, which could complicate the identification of ligation products. Secondary structures of both fragments were previously studied in our group and the locations of “fragile” sites, most susceptible to cleavage-inducing agents, in these fragments have been detected (shown as arrows in Figure 1) [13–15]. Figure 1 depicts secondary structures of HIV-RNA and M2-RNA.

Non-enzymatic reactions investigated in the present work are coupled, i.e. both cleavage and ligation stages are performed within a single reaction system under permanent thermal and chemical conditions. Based on our previous data [11,12], we assumed that cleavage step preceded the ligation. Upon cleavage, partially degraded RNA fragments are formed; supposedly these fragments are more susceptible to ligation than full-length RNAs due to lesser spatial hindrance and simpler secondary structure.

Cleavage and ligation reactions, occurring in solution containing only magnesium ions and RNAs may lead to the formation of a wide range of recombinant molecules. Therefore, it was essential to govern this reaction to yield a limited number of ligation products and, hereby, to imitate the primitive form of prebiotic selection *in vitro*. Toward this end, oligodeoxyribonucleotide complementary with its 5′-part to 13-mer region in HIV-RNA, and with its 3′-part—to 12-mer region within M2-RNA, was used as a template in the recombination reaction. Choice of DNA as a template was attributed to its inability to undergo transesterification reactions due to the absence of 2′-OH-groups. DNA:RNA duplexes adapt an A-form helix, similar to a structure of RNA:RNA duplexes. Therefore the use of chimeric complementary complexes was not expected to significantly alter the mechanism of reactions occuring in a pool of RNA molecules [17].

### 2.2. Reaction scheme and conditions

**Figure 2.**
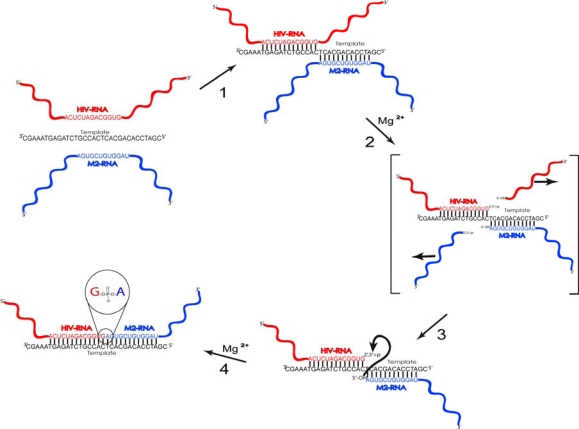
Template-directed non-enzymatic cleavage/ligation (recombination) reaction. 1) Formation of a pseudoduplex between HIV-RNA (left-hand substrate), M2-RNA (right-hand substrate) and the ON-template. 2) Cleavage of HIV-RNA and M2-RNA at “fragile” sites outside the pseudoduplex RNAs:template, with formation of terminal 2′,3′-cyclophosphates and 5′-OH. 3) and 4) Ligation within complementary complex of RNAs with the template in the presence of Mg^2+^. Formation of new phosphodiester bond between the fragments of HIV-RNA and M2-RNA. Arrows show the sequence of processes taking place in the reaction system.

We assume that in the reaction mixture containing both RNAs, oligodeoxyribonucleotide template (ON-template) and Mg^2+^ (the catalyst of cleavage/ligation reaction), at first, equilibrium will be achieved between free RNAs and template-bound RNA, one or both. This is a rapid process as compared to slow cleavage and ligation reactions [18–20]. In the pseudoduplex of RNAs with ONtemplate (step 1 in Figure 2) long dangling ends of both RNAs will be formed. Presumable scheme of processes resulting in a formation of novel RNA molecules is shown in Figure 2. It is well known that RNA within single-stranded regions is much more susceptible to transesterification (cleavage) than that within the complementary complex with another strand of RNA or DNA [21]. As a result of cleavage, 2′,3′-cyclophosphate is formed at the 3′-end of the left-hand substrate, and 5′-OH at the 5′- end of the right-hand substrate (step 2 in Figure 2). Then, nucleophilic 5′-OH-group attacks adjacent 2′,3′-cyclophosphate, initiating the second step of transesterification (ligation) and formation of a new phosphodiester bond. As a result of these processes, RNA molecule with novel sequence emerges, as ligating fragments differ from those formed at the cleavage step of recombination.

Cleavage/ligation conditions were similar to those we applied in our previous studies [11,12]. Reaction mixtures containing equimolar mixture of both RNAs, twofold molar excess of a template and 5 mM MgCl_2_, were incubated at pH 8.0, 37 °C during 3 days. After the incubation, Mg^2+^ ions were removed *via* binding with an equal molar quantity of EDTA, and nucleic acids were precipitated with ethanol. Obtained pool of RNAs was used for identification of the recombination products.

### 2.3. Identification of products of RNAs recombination

#### 2.3.1. RT-PCR

Due to additional spatial hindrance, the yield of template-directed recombination of the two 96-mer RNAs is lower than that in Mg^2+^-catalyzed cleavage/ligation reaction occurring between two short oligoribonucleotides [11]. Therefore the use of routine methods of detection of ligation products, e.g. radioactive labelling of RNA and visualization of obtained products *via* radioautographing of gels, was not considered as an optimal choice for the elaborated reaction system. For this reason, an indirect approach, RT-PCR, that enabled us to detect a selected range of recombinant products and finally to unravel mechanisms leading to their formation, was applied.

**Figure 3.**
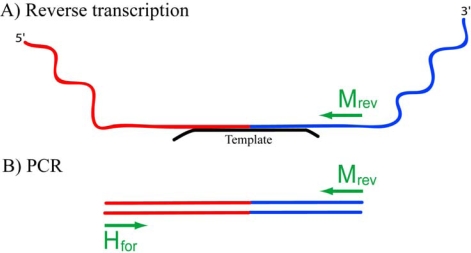
First step of identification of recombinant RNAs. A) Reverse transcription reaction with a primer M_rev_. B) PCR with primers H_for_ and M_rev_.

Reverse transcription was performed with the use of sequence-specific primer M_rev_ (Figure 3, A). The usage of M_rev_ primer for the reverse transcription restricts the pool of amplified recombination products by those where M2-RNA fragments comprising M_rev_ binding sites were the right-hand 5′-OH bearing substrates upon ligation step (see Figure 2, steps 3 and 4). In order to avoid elongation of the ON-template on reverse transcription step, it was designed to bear 5 nts and 2 nts overhangs at its 5′ and 3′ ends, respectively. Such modifications also facilitate the displacement of oligodeoxyribonucleotide from DNA:RNA complex by DNA polymerase. Control experiments confirmed that these overhangs effectively prevented the template from elongation on RT and PCR steps (primary data not shown).

PCR was performed using two pairs of primers: M_for_ / M_rev_ to synthesize the dsDNA fragment corresponding to M2-RNA, and H_for_ / M_rev_ for amplification of recombination molecules, formed in the non-enzymatic ligation reaction between 5′-OH-bearing M2-RNA fragments and 2′,3′-cyclophosphate-possessing fragments of HIV-RNA (Figure 3, B). Once ligation of such fragments had occurred in butt-to-butt manner, the product of amplification of recombinant RNA had to be 55 nts long (Figure 3, B). Binding sites of forward (H_for_) and reverse (M_rev_) primers were chosen not to overlap the template-binding site in order to ensure a valid amplification of the selected range of recombinant products.

**Figure 4.**
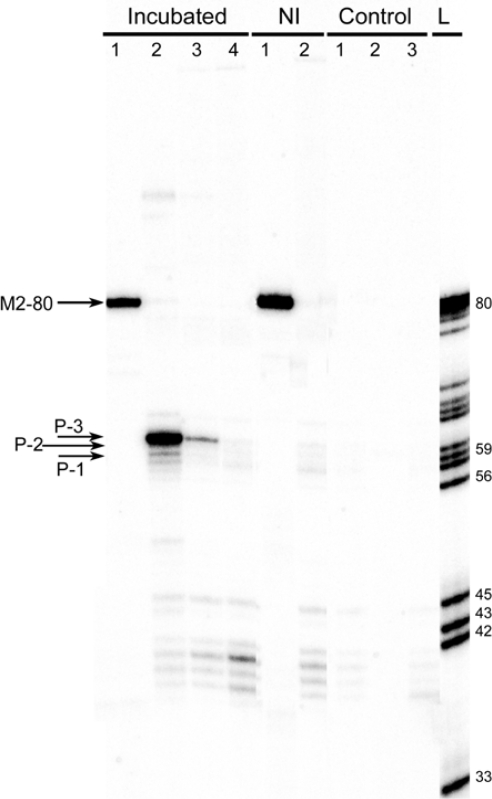
RT-PCR analysis of the products of template-directed recombination of M2- RNA and HIV-RNA. Autoradiogram of a 10% denaturing PAAG. “Incubated” group of lanes: RT-PCR analysis of reaction mixtures incubated under conditions of cleavage/ligation reaction at 37 °C for 3 days. 1) positive control (PCR with primers M_for_ and M_rev_); 2), 3), 4): PCR with primers H_for_ and M_rev_, using dilutions of cDNA mixture 1:1, 1:10^−3^ and 1:10^−6^, respectively. “NI” group: reaction mixture without incubation was used for RT-PCR analysis. 1) positive control (PCR with primers M_for_ and M_rev_); 2) PCR with primers H_for_ and M_rev_, and non-diluted cDNA mixture. “Control” group: RT-PCR was performed in the absence of RNA (1) or in the absence of cDNA (2 and 3) with PCR primers H_for_ and M_rev_ (1 and 2) and M_for_ and M_rev_ (3). “L”: DNA ladder (product of partial hydrolysis of M2–80 PCR product at adenine and guanine sites). Location of major products in gel is marked with arrows. M_rev_ primer was 5′-^32^P-labelled in all reaction mixtures.

PCR was performed in parallel reactions with 5′-^32^P-labelled (for analytical purposes) and unlabelled M_rev_ primer. In analytical series, cDNA mixtures in dilutions 1:1, 1:10^−3^ and 1:10^−6^, and 28 cycles of amplification in PCR were used. For a positive PCR control, we used primers M_for_ and M_rev_, and the same cDNA mixture in dilution 1:10^−3^, as M2-RNA should be presented in the ligation mixture in a relatively high concentration. Negative controls inferred PCR reactions under 3 conditions: 1) PCR with primers H_for_ and M_rev_, of the RT-mixture obtained in the absence of RNAs; 2) PCR with primers H_for_ and M_rev_ without a cDNA; 3) PCR in the absence of cDNA, with primers M_for_ and M_rev_. Radioactive amplicons were applied on 10% dPAAG. A product of partial cleavage of M2–80 radioactive PCR product (primers M_for_ and 5′-^32^P-M_rev_) at adenine and guanine sites was used as a ladder (Figure 4).

As follows form the figure, no products were amplified in negative control mixtures (“Control group”, lanes 1–3, and “NI” group, lane 2), and only the amplicon of M2-RNA was observed in a positive control experiment (lane 1 in “NI” and “Incubated” groups). The minor bands not exceeding 45 bp in length which are observed in the lanes of both incubated and non-incubated mixtures and seem not to depend on the dilution of cDNA, could arise from some nonspecific amplification upon PCR in the lack of a valid template. The data displayed in Figure 4 show that one major product (P3) and at least two minor products (P1, P2) are amplified upon using H_for_ and M_rev_ primers (“Incubated group”, lanes 2 and 3). The lengths of detected products, though they were close to the length of initially proposed recombinant product (55 nts), varied in the range of 1–5 nucleotides, indicating that actual recombination scheme is more complicated than it was initially predicted. Therefore, nucleotide sequencing of amplicons was undertaken for elucidating the underlying mechanisms of the events resulted to the the formation of such a variety of products.

In order to obtain the sequences of individual products, we used TA molecular cloning, based on a feature of *Taq* DNA polymerase, used in PCR, to synthesize sticky mononucleotide 3′-A-ends on each strand of PCR product.

#### 2.3.2. Molecular cloning

Each amplicon formed in RT-PCR regardless of its length and nucleotide sequence possesses two sticky ends, and thus may be inserted into T-vector using standard cloning approach. T-vector is a linearized form of 2.9 kbp long PTZ57R/T plasmid, bearing sticky 3′-ddT mononucleotide ends, required for recognition of amplicons and their incorporation into the T-vector by T4 DNA ligase. Apart from amplicon insert, the vector contains a gene of ampicillin resistance (Ap^r^) for further selection of bacterial cells transformed with this sort of plasmids.

In this work, we cloned a pool of RT-PCR products obtained under conditions corresponding to lane 2, “Incubated group” in Figure 4, into the T-vector. *E. coli* cells were transformed with a mixture of plasmids containing different TA-inserts and plated at ampicillin selective medium. Cells within each colony contained plasmids with the only type of TA-insert. Hereby we reached a separation of amplicons corresponding to different products of non-enzymatic recombination.

**Figure 5.**
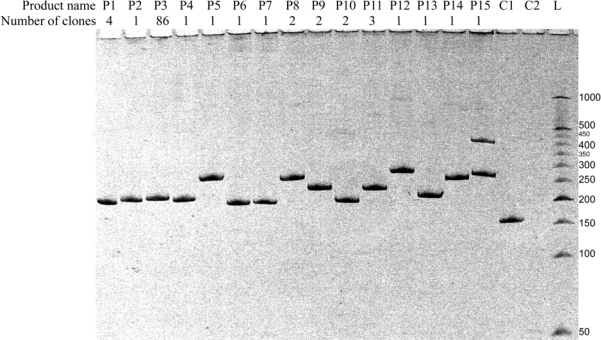
Bacterial colonies screening. Representative electrophoresis showing all detected types of products of RNA recombination. Lanes 1–15: products of bacterial colony PCR, amplified with the use of primers M13/pUC_for and M13/pUC_rev. C1 – positive control (PCR from circular plasmid without TA-insert), C2 – negative control (PCR without template), L – double-stranded DNA ladder. Figures below lane numbers show the number of colonies (clones) containing each type of TA-insert.

Bacterial colonies were screened and divided into groups on the basis of the length of TA-insert comprised within T-vectors using a bacterial colony PCR. Pieces of bacterial colonies were placed into PCR mixtures and their amplification was performed using standard M13/pUC_for and M13/pUC_rev primers, directed to plasmid regions located in 50–100 bp distance from the insert site. Obtained amplicons consisted of the insert (variable length) and plasmid region (permanent length, equal to 154 nts). Along with PCR performed on bacterial colony material, we made PCR from a circular plasmid without an insert and PCR without a template as positive and negative controls, respectively. All PCR products were separated in 8% dPAAG and visualized by staining with ethidium bromide. Analysis of the products’ lengths revealed 15 types of amplicons. From one to eight bacterial colonies representing each group of PCR inserts were selected for sequencing (30 colonies in total). Representative electrophoresis displaying different types of the products detected *via* bacterial colony PCR is shown in Figure 5.

As shown in Figure 5, products of 15 different types are detected in the mixture of recombinant RNAs. Five of them (P1–P4, P13) have lengths close to that of the expected butt-to-butt ligation product (Figure 5, lanes of the same names). The lengths of other ten products vary from 38 to 119 nts (Figure 5, lanes P5–P12, P14, P15). Numbers of screened colonies corresponding to each of the recombinant product type are different (Figure 5, row “number of clones”). Results of bacterial screening demonstrate that the reaction mixture contains one major product of recombination (P3, 86 colonies), two products obtained with a significantly lower yield (P1 and P11, four and three clones respectively), and 12 minor recombination products (1–2 clones for each). One colony contains two types of TA-inserts (P15) due to transformation of its parent cell with plasmids possessing different types of TA-inserts. Bacterial colony PCR releases more variety of products than that identified by RT-PCR with labelled primer (see Figure 4), which can be attributed to a higher sensitivity of the TA-cloning detection method in comparison to RT-PCR. It is notable that there is a correlation between the data obtained using these two methods of detection: ratio of quantities of products P1, P2 and P3 is the same for both detection strategies applied (compare Figure 4 and Figure 5). The diversity of different products with similar lengths necessitated the nucleotide sequencing of TA-inserts in the bacterial colonies representing each type of recombinant product.

#### 2.3.3. Sequencing of TA-inserts

For sequencing, from one to eight colonies from each of 15 groups of clones were selected. Eight colonies from the group P3 (out of total 86), and all the colonies from other, minor groups (1–4 clones) were subjected to sequencing. For this purpose, night cultures in LB+Ap medium were prepared from selected colonies, and plasmids were isolated from them using Sigma GeneElute^TM^ Plasmid Miniprep Kit. The plasmids were subjected to Sanger reaction, performed using primer M13/pUC_rev. Obtained sequences were aligned with initial HIV-RNA and M2-RNA, and with a sequence of the ON-template. Table 1 summarizes the results of TA-insert sequencing.

The data in the Table 1 demonstrates that at least 96 clones out of total 108 detected by bacterial colony PCR are the products of template-dependent recombination. Nevertheless, only four of them correspond to the products of butt-to-butt ligation, expected to be the major ligation strategy in the elaborated reaction system (P1).

**Figure 6.**
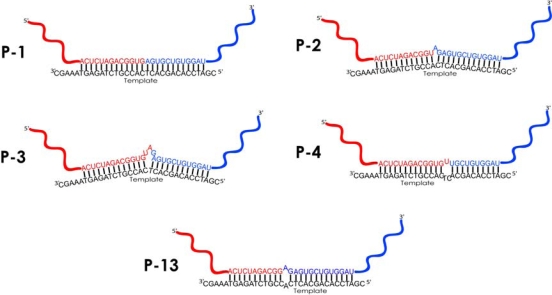
Products of template-governed recombination of RNAs. Ligation takes place within a full complementary complex of RNAs with a template (in butt-to-butt manner, P1), in RNA bulges of a size 1 or 3 nucleotides (P2, P3), in symmetric (P13) and asymmetric (P4) internal loops. Fragments of HIV-RNA are shown in red, of M2-RNA—in blue, ON-template is shown in black.

The majority of clones (86) contains an insert with a P3 type of amplicon, where the ligation is template-governed, but occurs in the 3 nts bulge loops of RNA formed at the ligation region. Other, minor products of template-governed recombination are also formed as a result of ligation in the loops of different size, formed both by RNA and the ON-template (see Figure 6 for structures): 1 nt bulge loop (P2, one clone); 3 nts bulge loop, with repeated sequence of H_for_ primer binding site (P11, three clones); symmetric 2 nts internal loop (P13, one clone) and asymmetric 3 nts internal loop (P4, one clone). Apart from the products of template-directed ligation, 9 types of products are formed due to ligation in random sites beyond the template-binding site (total 12 clones). Interestingly, two types of products, P6 and P15, appeared as a result of ligation within two or three sites, and exterior two-nucleotide fragments were inserted in HIV- and M2-RNA ligating fragments. As far as our experiments aimed in the investigation of template-guided recombination processes, we accentuate on discussion of only this sort of recombinant products.

### 2.4. Discussion

To design the model system for our investigation, we proceeded from the assumption that using ON-template facilitates predominant synthesis of one particular product by approaching RNA fragments in butt-to-butt manner. However, the ligation reaction occurs mostly within another complexes, part of which is formed with an involvement of a template, and another one is presumably template-independent.

In our previous work, we investigated the template-dependent ligation reaction occuring in the complex of two oligonucleotides with tetraadenylate dangling ends whose cleavage, induced by magnesium ions, resulted in formation of a pseudo-duplex favoring formation of one type of the ligation product [11]. In the current study, we have found i) wide range of products of RNA recombination; ii) preferred ligation within the bulge loops (structures P2, P3, P4 and P13 in Figure 6) and iii) formation of template-independent recombination products, though with a low yield (12 of 108 clones).

There are several factors explaining the range of recombinant products formed. Firstly, Mg^2+^-induced cleavage occurs at various sites of RNA. According to the previously obtained data on the cleavage profiles of given RNA substrates, there are at least 12 sites in HIV-RNA molecule, which are likely to be cleaved under the conditions we use in this study, and at least 8 sites in M2-RNA substrate (see Figure 1) [13–15]. Secondary cleavages multiply the number of products. Thus, cleavage reactions result in formation of a variety of RNA fragments of different lengths, capable of undergoing non-enzymatic ligation reaction.

Secondly, binding of ON-template to RNA favors the cleavage within RNA regions adjacent to the formed duplex, and protects bound region, thus ensuring the “survival” of fragments containing template-binding site and their posterior participation in ligation reactions [13].

Thirdly, conformational preferences of recombination reactions play crucial role in selection of the products at both cleavage and ligation stages. Little is known about the conformational features of processes occurring in ligation reactions [9,22,23]; however, principles ensuring cleavage are very similar to those for ligation, and may be described in the same terms. Dependence of transesterification reaction efficacy on conformation of RNA backbone was marked out in a number of works [24,25]. Susceptibility of internucleotide linkages to non-enzymatic cleavage depends on disposition of attacking 2′-OH group and leaving 5′-O-group. The most rapid cleavage occurs in so-called “in-line” conformation, wherein the attacking nucleophile, the leaving group and the phosphorus atom form an angle close to 180° [8,21,26]. Flexibility of ribose phosphate backbone in RNA loops is higher than that in stem regions, ensuring the ease of formation of optimal spatial conformations between the active groups (2′,3′-cyclophosphate and 5′-OH). It was reported that within single-nucleotide bulges the bulged nucleotide may attain both looped-out and stacked-in positions. Regardless of the position, the angles defined by 2′-O-P-5′-O are close to 180°, which fits the best to in-line displacement, in contrast to 67° observed for the regular A-type helix [21]. Thus, occurrence of ligation within single-nucleotide bulge loop in the product P2 may be explained with attainment by ribose phosphate backbone of a proper local conformation.

Other evidence for in-line displacement of ligating groups within RNA bulges and loops is found in some properties of the non-enzymatic polymerization reaction. In the absence of chemical activating agents template-directed non-enzymatic polymerization of ribonucleotides almost invariably results in production of the unnatural 2′,5′-isomer. The constraint of the double helix forces the formation of the 2′,5′-bond from the cyclic phosphate [27]. Similar data was obtained in our previous work on investigation of template-directed ligation of oligonucleotides, where up to 95% of newly formed bonds were 2′,5′ in nature [12]. In contrast, different conformational arrangements are possible for nucleotides in bulges, depending on the type of base in the unpaired nucleotides and in the flanking base pairs. Ultimately these arrangements define the local flexibility of ribose phosphate backbone and its predisposition to pseudorotation in the cleavage or ligation site [21,28]. In case of ligation within bulges or internal loops, short (1–2 nts) dangling ends of RNA fragments, converged by the template, seem to form spatial structures optimal for ligation to occur. Our results on preferable formation of new phosphodiester bonds within bulge loops 3 nts in size is in accordance with existing data that bulges of this sort do not tense the ribose phosphate backbone of RNA significantly [29,30].

Some recombinant products detected in the ligation mixture contain symmetric and asymmetric 2–3 nts internal loops (P4 and P13). According to the literature data, the occurrence of internal loops of this size does not significantly disturb the continuous stacking of nucleotides in a duplex. Meanwhile, the nucleotides of the internal loop have more freedom and hence larger entropy than in the double helix, but smaller entropy than in the single strands, as it is flanked by base-pair closures at each end [31]. According to the assumptions made by Gralla *et al*., the loop size of two or three nucleotides is sufficient for ensuring the local flexibility of a ribose phosphate backbone essential for ligation to occur, but still does not disrupt the structure of a double helix dramatically [31]. Apart from the products of template-governed ligation discussed above, we observed the formation of nine other products (P5—P10, P12, P14, P15). Routine alignment of sequences of these products with the sequence of the ON-template does not provide an evidence of the role of template in their formation. These products are poorly presented in the obtained pool of clones (12 clones out of total 108). Two possible hypotheses can be employed to explain their formation. The first one is an occasional convergence of two different molecules followed by ligation, as it was suggested by Chetverin *et al*. Those reactions were reported to undergo one of two mechanisms of transesterification depending on the reaction conditions: i) through the attack of an internucleotide phosphodiester bond with a 3′ hydroxyl group of the 5′ fragment, or ii) *via* formation of an intermediate bearing 2′,3′-cyclophosphate, subsequently attacked by 5′-OH group, as was described in p. 2.2 [9,10]. Another explanation lays in possible involvement of M2- or HIV-RNA molecules in recombination reactions both as substrates and templates governing the ligation in unpredicted sites. In this case, like in template-directed reactions described above, Watson-Crick base pairing may be considered as a mechanism for approaching the active ends of two molecules (2′,3′-cyclophosphate and 5′-OH), which leads to formation of a new phosphodiester bond. These assumptions are to be proved later through the use of a more representative sample and search of the regions of homology between RNAs forming the minor products of recombination. That requires the implementation of advanced alignment strategies, and is considered as a separate task.

### 2.5. Limitations of the detection method

Apparently, amplification of ligation products in the used experimental model was limited to those molecules which contained both H_for_ and M_rev_ primer-binding sites. This method of detection was aimed to investigate the possibility of the template-directed ligation, which occurs in the detected area of recombinant RNA. The scheme used does not allow the detection of products whose ligation sites are located out of amplifying region restrained by primer binding sites. It also leaves invisible the products constructed of 5′-part of M2-RNA and 3′-part of HIV-RNA. Another limitation consists in the inability to detect the nature of newly formed phosphodiester bonds. M-MuLV DNA polymerase used in reverse transcription reaction is able to read through both 3′,5′- and 2′,5′-bonds, although the latter are processed with a lower rate [32]. Therefore we discuss only sequences and secondary structures of recombinant RNAs in the ligation sites, and do not accentuate the chemical nature of phosphodiesters. However, these limitations were essential to dispose of the minor products and come up with the reliable description of mechanisms leading to the formation of the major products.

## 3. Experimental Section

### 3.1. Enzymes and chemicals

[γ-^32^P]-ATP (specific activity >3000 Ci/mmol) was from Biosan Co. (Russia). Enzymes: calf intestinal alkaline phosphatase and endonuclease Fok I were purchased from “SibEnzyme”, Russia. T7 RNA polymerase, *Taq* DNA polymerase, M-MuLV DNA polymerase were produced in this Institute. T4 polynucleotide kinase was from Fermentas. TA cloning was proceeded using “InsTAclone^TM^ PCR Cloning Kit” (Fermentas). Plasmid DNA was isolated with “GenElute^TM^ Plasmid Miniprep Kit” (Sigma). BigDye v.1.1 sequencing mixture was from Applied Biosystems. All the chemicals were of analytical or ACS grade, and autoclaved Milli-Q^TM^ water was used for all procedures. M2-RNA of influenza virus was synthesized by T7 transcription from plasmid pSVK3M2, this Institute collection. HIV-1 fragment was obtained *via* transcription of plasmid pHIV-2, kindly provided by Prof. H.J. Gross (University of Wurzburg, Germany).

### 3.2. Oligonucleotides

Oligonucleotides were synthesized using standard automated solid-phase methods and purified by reverse-phase liquid chromatography in this Institute. Primers were as follows: M2–96_for_ – ACAAGCTTTAATACGACTCACTATAGGGCCTTCTACGGAAGGAGTACC (T7 promoter region is underlined), M2–96_rev_ – CGAGACAAAATGACTGTCGTCAGC, M_for_ – CTACGGAAGGAGTACC TGA, M_rev_ – ATGACTGTCGTCAGCATC, H_for_ – GAGATCCCTCAGACCACT, M13/pUC_for – GTAAAACGACGGCCAGT, M13/pUC_rev – CAGGAAACAGCTATGAC. ON-template – CGATCCACAGCACTCACCGTCTAGAGTAAAGC (overhangs are underlined).

### 3.3. 5′-^32^P-oligonucleotide labeling

Primer M_rev_ was 5′-end labeled with ^32^P using T4 polynucleotide kinase and [γ-^32^P]-ATP. A reaction system (total volume 10 μL) containing 300 pmol of oligonucleotide, ligation buffer, 10 u of polynucleotide kinase and 0.1 mCi [γ-^32^P]-ATP was incubated at 37 °C for 1 hour. Then labeled oligonucleotide was purified in 20% denaturing polyacrylamide gel (dPAAG).

### 3.4. Plasmid linearization

Plasmid pHIV-2 was linearized with Fok I endonuclease according to the manufacturer’s manual. Reaction mixture of a total volume 200 μL, containing 40 μg of plasmid DNA, restriction buffer and 120 u of endonuclease, was incubated at 37 °C for 4 hours. Then mixture was extracted consecutively with solution TE-phenol-chloroform (1:1) and chloroform-isoamyl alcohol (24:1). DNA from the aqueous phase was precipitated with ethanol and dissolved in water.

### 3.5. Amplification of M2-DNA fragment

M2-DNA fragment was amplified from the plasmid pSVK3M2 using primers M2–96_for_ and M2–96_rev_. PCR mixture contained 67 mM Tris-HCl (pH 8.0), 16 mM (NH_4_)_2_SO_4_, 1.5 mM MgCl_2_, 0.01% Tween-20, 0.2 mM dNTPs, 1.3 μM each PCR primer, 0.5 μg/mL plasmid and 5 u of *Taq* DNA polymerase in a final volume of 50 μL. PCR was performed in Hybaid PCR Express thermal cycler, starting with 3 min of preincubation at 95 °C, and followed by 30 amplification cycles (1 min, 94 °C; 1 min, 52 °C; 1 min, 72 °C). Amplification product (121 bp) was purified by phenol extraction (TEphenol : chloroform (1:1), then chloroform only) and precipitated from the aqueous phase with ethanol. Dissolved in water precipitates were used as templates for T7 transcription.

### 3.6. T7 transcription

We applied T7 transcription for the synthesis of M2-RNA and HIV-RNA from M2-DNA (obtained by amplification of pSVK3M2 plasmid fragment) and linearized plasmid pHIV-2, respectively. Reaction mixtures of total volume 100 μL, containing 40 mM Tris-HCl (pH 8.1), 16 mM MgCl_2_, 2 mM spermidine, 4 mM NTPs, 5 mM DTT, 0.2 mg/mL BSA, 0.6 mg/mL of DNA and 480 u of T7 polymerase, were incubated at 37 °C for 4 hours. After incubation, RNA was purified by phenol extraction [phenol-chloroform (1:1), then chloroform-isoamyl alcohol (24:1)], followed by purification in 8% native PAAG. The visualized band was cut out and RNA was eluted from the gel with 3 mL of elution solution (0.6 M NH_4_Ac (pH 5.0), 0.1 mM EDTA, 0.1% SDS) on a shaker at 4 °C for 4 hours. Eluted RNA was precipitated with ethanol and dissolved in water.

### 3.7. Dephosphorylation of RNA

A reaction mixture (total volume 50 μL) containing 8 μg of M2-RNA or HIV-RNA, 50 mM bis(tris(oxymethyl)methylamino)propane HCl buffer (pH 8.0), 2% formamide, 0.2% SDS, 1.25 mM DTT and 1.5 u of Calf intestinal alkaline phosphatase (CIP) was incubated at 37 °C for 45 minutes. Then 1.5 u of CIP was added again to the reaction mixture and it was additionally incubated under the same conditions. After incubation, mixture was subjected to twofold extraction with phenolchloroform (1:1), then chloroform-isoamyl alcohol (24:1). RNA was precipitated from the aqueous phase by 95% ethanol, washed twice with 75% ethanol, dissolved in water and stored at −20 °C.

### 3.8. Non-enzymatic cleavage/ligation reaction

Reaction mixtures (total volumes 30 μL) containing bis(tris(oxymethyl)methylamino)propane HCl buffer (pH 7.5, 8.0, 8.5), 0.5 μM dephosphorylated M2-RNA and HIV-RNA, 1.0 μM of the ONtemplate (in template-directed reactions) and 5 mM MgCl_2_, were incubated at 37 °C for 3 days. After incubation, EDTA was added to the ligation mixtures in a concentration equal to that of MgCl_2_. Volumes of all probes were adjusted to 100 μL with water, and RNA was precipitated with ethanol in the presence of 0.3 M NaAc and 10 μg of glycogen. Precipitates were washed with ethanol, dissolved in 15 μL of water and used in reverse transcription reaction.

### 3.9. Reverse transcription and PCR of ligation products

The DNA template for PCR amplification was produced from the ligation mixture of recombinant RNA in reverse transcription reaction. A reaction mixture with an initial volume of 12 μL, containing 1.25 μM of specific reverse primer and 1 μL of mixture of products of the cleavage/ligation reaction (see p. 3.8), was subjected to denaturation at 70 °C for 5 min and then chilled on ice for 3 min. After that NTPs of a final concentration 1 mM and M-MuLV buffer were added, volume was adjusted to 18 μL with water, and mixtures were incubated at 37 °C for 5 min. Finally, 20 u of M-MuLV polymerase were added and the mixture of a final volume 20 μL was incubated at 42 °C for 60 min. The reaction was terminated by heating at 70 °C for 10 min. Obtained cDNA was directly used in PCR. PCR was performed from reverse transcription (RT) products using primers H_for_ and M_rev_. In a positive control (to confirm M2-RNA presence in the reaction mixture) primers M_for_ and M_rev_ were used. PCR mixtures contained 67 mM Tris-HCl (pH 8.0), 16 mM (NH_4_)_2_SO_4_, 1.5 mM MgCl_2_, 0.01% Tween-20, 0.25 mM dNTPs, 0.8 μM each PCR primer, 1 μL of RT-product in dilutions 1:1, 1:10^−3^, 1:10^−6^ and 2 u of *Taq* DNA polymerase in a total volume of 20 μL. PCR was started with 3 min of preincubation at 95 °C and followed by 25 cycles of amplification (1 min, 94 °C; 1 min, 56 °C; 1 min, 72 °C). Final cycle contained prolonged elongation step (72 °C, 10 min) for complete formation of sticky A-ends. PCR products were used directly for TA cloning. In parallel, we run PCR with 5′-^32^P-labeled M_rev_ primers for visualization purposes. In this case aliquots of PCR products were mixed with loading buffer and applied on 10% dPAAG electrophoresis. Visualization was accomplished by exposing dried gels on FX Imaging Screen, followed by screen scanning on Molecular Imager FX-PRO Plus (Bio-Rad) and images processing in Quantity One v.4.2.3 Software (Bio-Rad). Amplification products were identified by electrophoresis on 10% dPAAG using DNA ladder, obtained *via* chemical cleavage of M2–80 radioactive PCR product (synthesized using primers M_for_ and 5′-^32^P-M_rev_, product length 80 bp) at adenine and guanine sites. Reaction mixture of a volume 30 μL, containing 10 μL of 5′-^32^P-labeled M2–80, and 20 μL of 3% diphenylamine solution in formic acid, was incubated at 37 °C for 5 and 10 minutes. At the end of incubation, 100 μL of water was added and reaction mixtures were subjected to twofold extraction with 300 μL of diethyl ether. DNA from the aqueous phase was precipitated with 10 volumes of 2% LiClO_4_ solution in acetone, pellets were washed with acetone, dissolved in water and then added to the loading solution.

### 3.10. TA cloning

The PCR products were cloned using the “InsTAclone^TM^ PCR Cloning Kit” (Fermentas), according to the manual provided by the supplier. Briefly, 0.54 pmol of PCR products, possessing sticky A-ends, was ligated with 0.18 pmol of linear plasmid vector pTZ57R/T, containing sticky ddT-ends, using T4 DNA ligase. The obtained recombinant plasmids were used for transformation of competent *E. coli* DH5α cells, prepared using the same Kit. Transformed cells were plated at LB agar Petri dishes with ampicillin at concentration of 50 μg/mL. Dishes were incubated at 37 °C for 20 hours and then stored at 4 °C. Plasmids were isolated using “GenElute^TM^ Plasmid Miniprep Kit” (Sigma) according to the manual of supplier. Briefly, night cultures (OD~0.5) were prepared from corresponding bacterial colonies in 3 mL of LB medium containing ampicillin at a concentration of 50 μg/mL. Cells were harvested by centrifugation, resuspended, lysed and then the solution was neutralized. Cell debris was precipitated by centrifugation, cleared lysate was loaded at microcentrifuge spin columns, and after washing plasmid DNA was eluted from the column with 100 μL of water. Isolated plasmids were used directly for sequencing purposes.

### 3.11. Bacterial colony PCR

Fragments of individual bacterial colonies were dissolved in 20 μL of PCR master mix, containing 67 mM Tris-HCl (pH 8.0), 16 mM (NH_4_)_2_SO_4_, 1.5 mM MgCl_2_, 0.01% Tween-20, 0.2 mM dNTPs, 0.3 μM of primers M13/pUC_for and M13/pUC_rev, and 2 u of *Taq* DNA polymerase. PCR was started with 3 min of preincubation at 95 °C and followed by 27 cycles of amplification (30 s, 94 °C; 30 s, 51 °C; 1 min, 72 °C). PCR products were analyzed in 8% dPAAG and visualized by staining of gels with ethidium bromide.

### 3.12. DNA sequencing

Sequencing of constructs possessing the DNA inserts was performed *via* Sanger sequencing reaction with mixture BigDye v.1.1 (Applied Biosystems) followed by further analysis of products at automatic gel analyzer ABI 3130xl (Applied Biosystems). For the sequencing reaction, 5 pmol of M13/pUC_rev primer, 200–400 fmol of plasmid, sequencing buffer and 1.5 μL of BigDye were mixed and adjusted to a final volume of 30 μL by adding water. The reaction was run according to the following program: 10 s preincubation at 96 °C; 3 cycles: 8 s, 96 °C; 4 min, 64 °C; 5 cycles: 8 s, 96 °C; 4 min, 60 °C; 17 cycles: 10 s, 96 °C; 5 s, 50 °C; 4 min, 60 °C; final step denaturation: 3 min, 96 °C. Products of sequencing reaction were purified from the unincorporated BigDye by precipitation with isopropyl alcohol, pellets were dried on SpeedVac evaporator for 3 minutes, dissolved in formaldehyde and subjected to analysis at gene analyzer. Sequences were determined in Sequence Scanner Software v. 1.0 (Applied Biosystems) and aligned with initial RNA sequences using Vector NTI v.10.0.1 Software (Invitrogen).

## 4. Conclusions

The non-enzymatic spontaneous recombination reactions discussed in this work were perfomed under very simple conditions, and, presumably, could be easily realized in prebiotic proteinless environment and lead to the formation of a pool of new RNA molecules. Notwithstanding the simplicity of chemical conditions, the processes taking place in the reaction system are very complicated and hardly predicted. It was found that nucleic acid template did not direct, but just facilitated the ligation, providing opportunity for approaching RNA dangling ends of different lengths and nucleotide sequences. RNA molecules appeared not to be prone to ligation within a fully complementary complex with a template, though some clones with this type of recombinant product were detected. Instead, the ligation occurred mostly in the elements of imperfect secondary structure of RNA, namely bulge loops of a size of one or three nucleotides and internal loops of a two or three nucleotide size. In spite of energetic preference of ligation within a duplex with the template, the predominant part of new phosphodiester bonds is formed in the 3 nts RNA bulges that strain spatial structure of RNA due to the double helix bend in the bulge area. However, local conformational preference for formation of this sort of products may be prevalent over thermodynamic stability of newly formed structures, providing an opportunity for occurrence of loop-bearing RNAs with novel sequences. Along with the products of template-governed ligation, we observed a number of products of template-independent ligation. However, formation of this sort of molecules may be attributed to the action of RNA substrates as templates for their self-ligation. If this is the case, proposed non-enzymatic mechanisms seem as a very plausible pathway for the formation, selection and evolution of first informational biopolymers in the prebiotic world.

## Figures and Tables

**Table 1.** Nucleotide sequences of products of HIV-RNA and M2-RNA recombination in the regions between H_for_ and M_rev_ primer binding sites.

Amplicon		Sequences of amplified regions of recombinant RNA molecules2)	Number of detected clones3)		Comments
Name1)	Length		Bact. colony PCR	Sequencing	
P1	55	[*H_FOR_*] CUAGACGGUGAGUGCUGUG [*M_REV_*]4)	4	4	Ligation in butt-to-butt manner^*^
P2	56	[*H_FOR_*] CUAGACGGUAGAGUGCUGUG [*M_REV_*]	1	1	1 nt RNA bulge loop^*^
P3	58	[*H_FOR_*] CUAGACGGUGUAGAGUGCUGUG [*M_REV_*]	86	8	3 nts RNA bulge loop^*^
P4	54	[*H_FOR_*] CUAGACGGUG***U***UGCUGUG [*M_REV_*]	1	1	Asymmetric 3 nts internal loop ^*^
P5	108	[*H_FOR_*] CUAGACGGUGUAAAAAUCUCUAGC AGUGGCGCCAUGAGGGAAGAAUAUCGAAAGGAA CAGCAGAGUGCUGUG [*M_REV_*]	1	1	Template-independent recombination
P6	38	[*H_FOR_*]AU[*M_REV_*]	1	1	
P7	39	[*H_FOR_*] CUA[*M_REV_*]	1	1	
P8	107	[*H_FOR_*] CUAGACGGUGUAAAAAUCUCUAGC AGUGGCGCAUGAGGGAAGAAUAUCGAAAGGAAC AGCAGAGUGCUGUG [*M_REV_*]	2	2	
P9	73	[*H_FOR_*] CUAGACGGUGUAUCGAAAGGAACA GCAGAGUGCUGUG [*M_REV_*]	2	2	
P10	42	[*H_FOR_*] CUAGAU[*M_REV_*]	2	2	
P11	76	[*H_FOR_*] [*H_FOR_*] CUAGACGGUGUAGAGUGCU GUG [*M_REV_*]	3	3	3 nts RNA bulge loop, double forward primer
P12	119	[*H_FOR_*] UAGACGGUGUAAAAAUCUCUAGCA GUGGCGCCCGAACAGGGACAUGAGGGAAGAAUA UCGAAAGGAACAGCAGAGUGCUGUG [*M_REV_*]	1	1	Template-independent recombination
P13	55	[*H_FOR_*] CUAGACGG***A***GAGUGCUGUG [*M_REV_*]	1	1	Symmetric 2 nts internal loop^*^
P14	105	[*H_FOR_*] CUAGACGGUGUAAAAAUCUCUAGC AGUGGCGCGAGGGAAGAAUAUCGAAAGGAACAG CAGAGUGCUGUG [*M_REV_*]	1	1	Template-independent recombination
P15	109	[*H_FOR_*] UAGACGGUGUAAAAAUCUCUAGCA GUGGCGCCCGAACAGGGACUUUAUCGGGAGGAA CAGCAGAGUGCUGUG [*M_REV_*]	1	1	Replacement of AA by GG (template-independent recombination)

* See Figure 6 for structures.

^1)^ Amplicon names correspond to those in Figure 5.

^2)^ Only RNA sequences between regions corresponding to primers H_for_ and M_rev_ are shown.

^3)^ Number of colonies, detected by bacterial colony PCR and *via* nucleotide sequencing of plasmids.

^4)^ RNA regions originating from HIV-RNA are shown in red, from M2-RNA—in blue. Template-binding regions are underlined; sequences corresponding to primers are shown as [*H_FOR_*] and [*M_REV_*]. RNA nucleotides within bulge loops are not underlined, nucleotides in internal loops and mismatches are shown in italic. External insertions are highlighted with yellow.
